# Acute Generalized Exanthematous Pustulosis in a Patient With Crohn’s Disease Being Treated for Streptococcal Pharyngitis and Sternoclavicular Joint Abscess

**DOI:** 10.7759/cureus.77595

**Published:** 2025-01-17

**Authors:** David Shi, Mona Ghias, Asif Haris, Kody Heubach

**Affiliations:** 1 Internal Medicine, West Virginia University, Morgantown, USA; 2 Pathology, Anatomy and Laboratory Medicine, West Virginia University, Morgantown, USA

**Keywords:** acute generalized exanthematous pustulosis (agep), adverse drug reaction, chrone's diseaes, pustular lesion, sternoclavicular abscess

## Abstract

Acute generalized exanthemous pustulosis (AGEP) is a rare skin reaction most commonly due to an adverse drug reaction. The rash is characterized by the sudden onset of diffuse pustules on a maculopapular rash. We present a case of a 62-year-old female treated with cephalexin for streptococcal pharyngitis who was admitted with the characteristic rash over her chest, abdomen, arms, and legs that started four days after starting the antibiotic. The diagnosis was further confirmed with a punch biopsy of the affected skin. The rash quickly resolved after discontinuing cephalexin. The patient’s course was complicated by findings of a left sternoclavicular joint abscess requiring surgical debridement and resection along with an extended course of intravenous antibiotics. It was felt the abscess was caused by hematogenous spread from the patient’s original streptococcal infection as opposed to being directly due to the AGEP, which further added to the complexity of the patient's case.

## Introduction

Acute generalized exanthematous pustulosis (AGEP) is a rare skin condition characterized by the sudden onset of diffuse pustules on a maculopapular rash [1,2]. AGEP is characterized by multiple punctate, sterile pustules and subsequent desquamation and is typically a drug reaction, most commonly secondary to beta-lactam antibiotics [3]. Here, we present a case of a patient with Crohn's disease who developed AGEP following the use of antibiotics for treatment of streptococcal pharyngitis whose course was further complicated by a sternoclavicular joint abscess.

## Case presentation

A 62-year-old female with a history of Crohn's disease presented to outpatient urgent care for severe throat pain. Her Crohn's disease was felt to be inactive, and she was not on immunosuppressive therapy. However, she did have a remote history of small bowel resection. The patient was diagnosed with streptococcal pharyngitis by rapid polymerase chain reaction (PCR) and subsequently started on cephalexin. 

**Figure 1:**
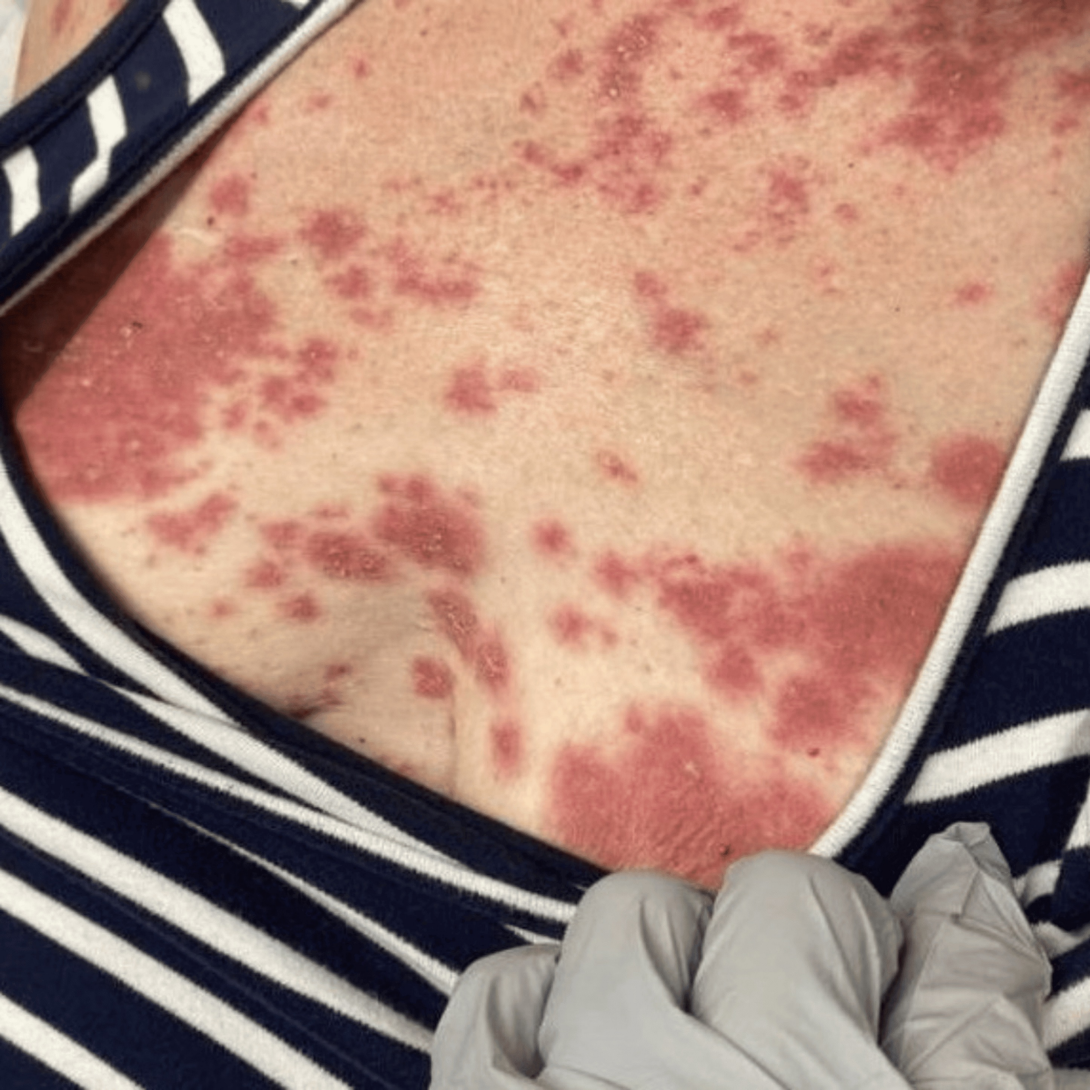
Erythematous pustular lesions over the chest.

Four days after her diagnosis, she presented to the emergency department after developing a diffuse maculopapular rash with pustules over her chest, abdomen, arms, and legs (Figure 1).

She also reported new onset neck and left shoulder pain. Laboratory values showed elevated white count and inflammatory markers, as detailed in Table 1. 

**Table 1:** Laboratory values during hospital course. WBC: white blood cells; HGB: haemoglobin; PMN: polymorphonuclear leukocyte; CRP: c-reactive protein.

Lab	Normal range	Value on admission	Value after four weeks
WBC	3.7-11.0x10^3 ^/uL	22.2x10^3 ^/uL	11.6x10^3 ^/uL
HGB	11.5-16.0 g/dl	13.4 g/dl	11.5 g/dl
Platelet	150-400x10^3 ^/uL	590x10^3 ^/uL	820x10^3 ^/uL
PMN’s	%	77%	69%
CRP	<8.0 mg/L	133.4 mg/L	13.5 mg/L
Blood culture 1^st^ set	Negative	Negative	Not Required
Blood culture 2^nd^ set	Negative	Negative	Not Required

**Figure 2:**
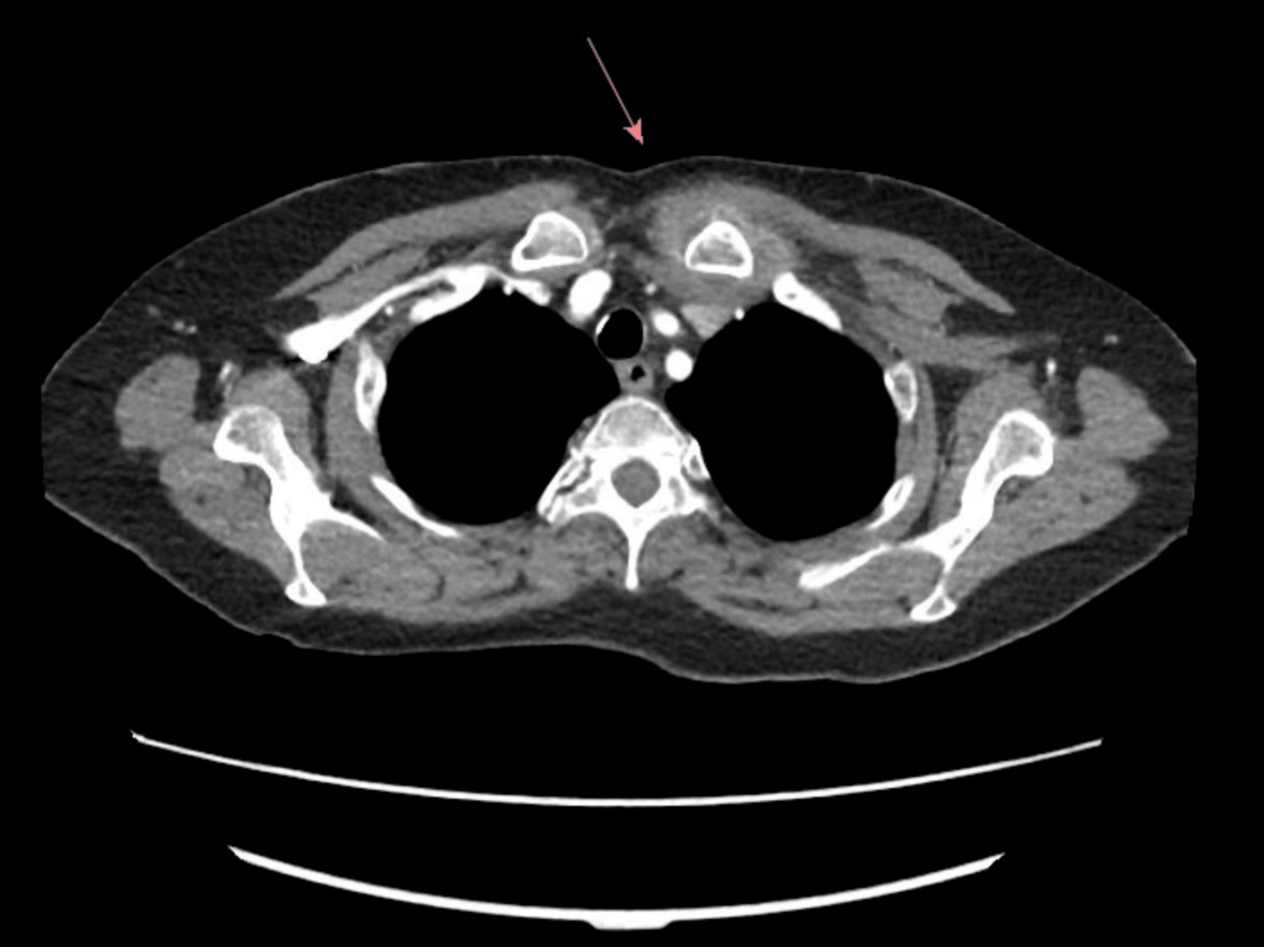
Low-density fluid collection measuring up to 2.7 cm primarily localized to the left sternoclavicular region. There is extension of stranding/inflammation in the pre-vascular space.

Computed tomography (CT) of the chest showed a 2.7 cm rim-enhancing fluid collection localized to the left sternoclavicular region (Figure 2). 

**Figure 3:**
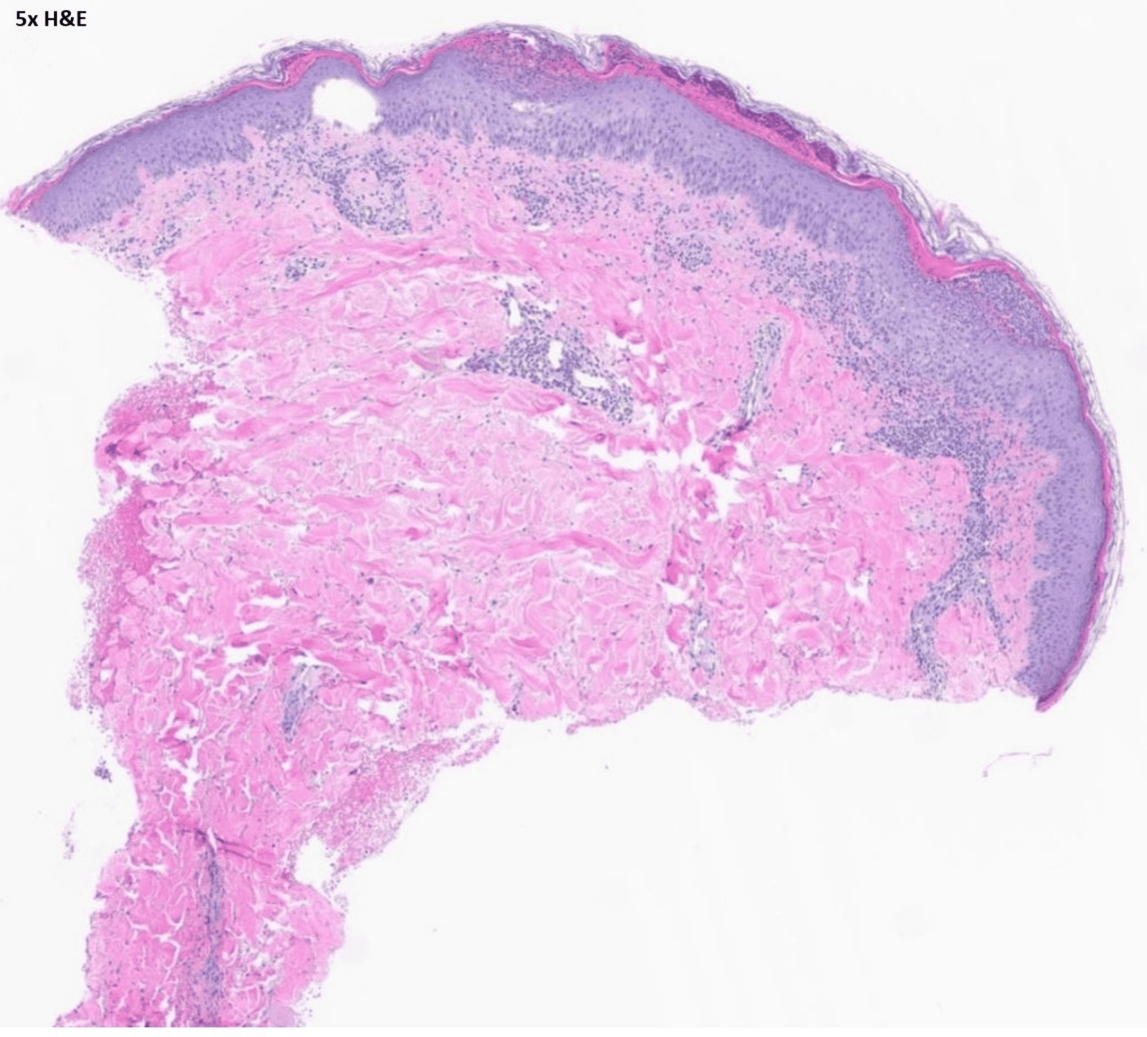
H and E stain under 5X magnification showing most of the punch biopsy with subcorneal pustules. H and E: hematoxylin and eosin stain.

**Figure 4:**
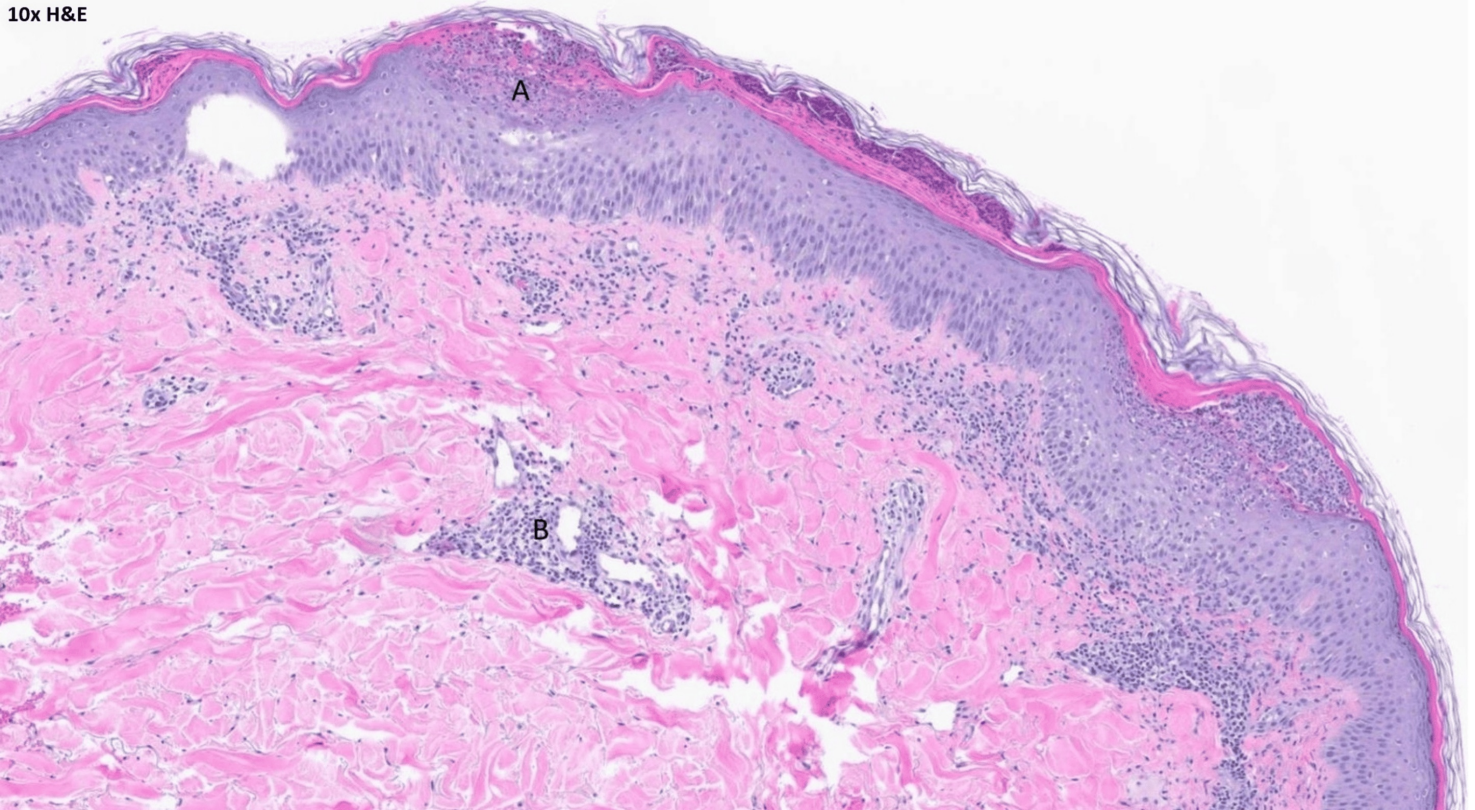
H and E stain under 10X magnification. (A) a better view of subcorneal pustules and presence of inflammatory cells. (B) there are perivascular lymphocytic infiltrates in the dermis. H and E: hematoxylin and eosin stain.

**Figure 5:**
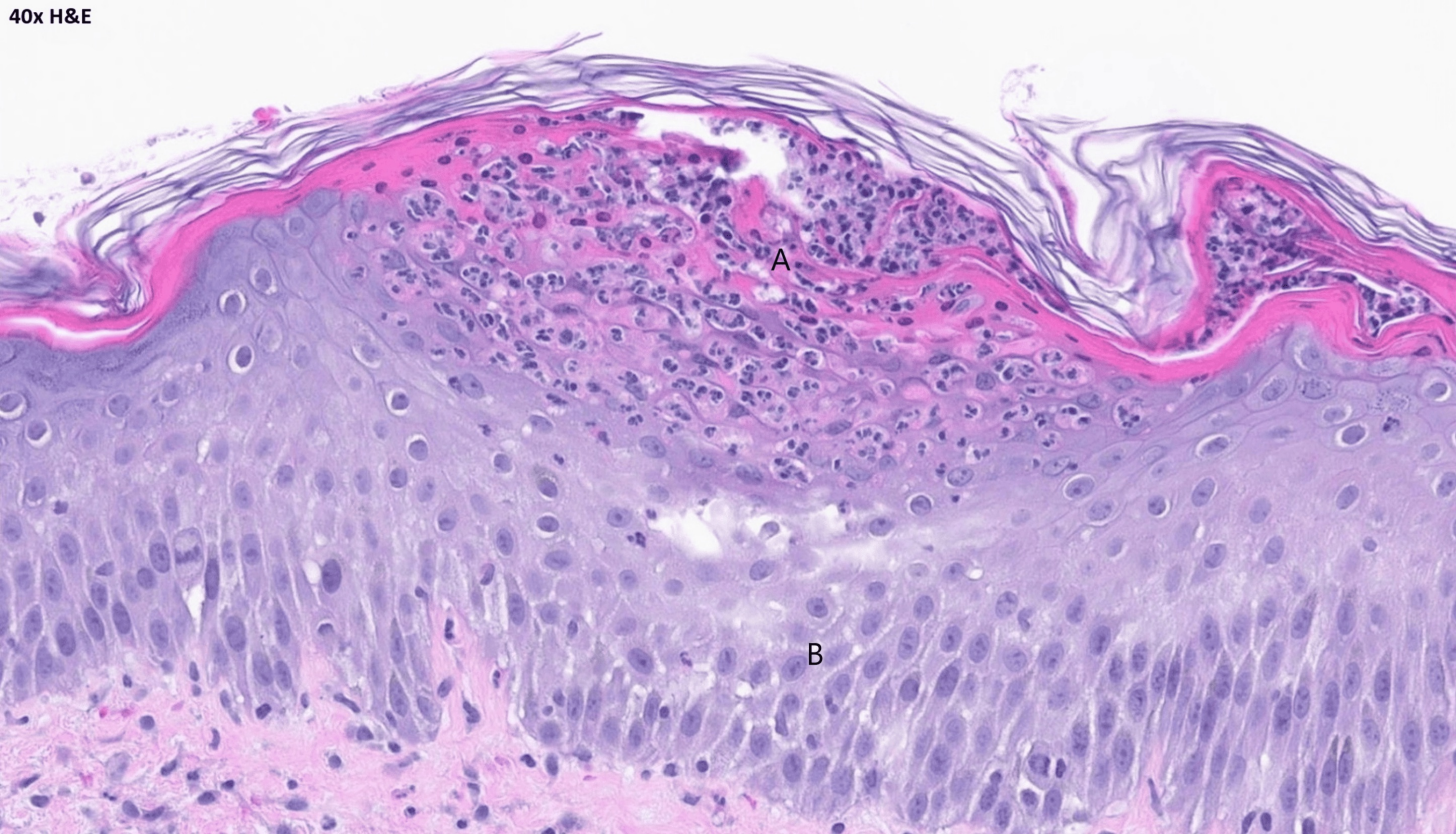
H and E stain under 40X magnification. (A) showing subcorneal collection with neutrophils. There are areas of spongiosis in the deeper portion of the epidermis. (B) eosinophils are seen in AGEP but are not essential for diagnosis. H and E: hematoxylin and eosin stain.

The patient was admitted to the hospital. Her cephalexin was immediately stopped, and her antibiotics were changed to daptomycin and aztreonam for broad-spectrum coverage. Dermatology evaluated the patient and recommended topical steroid therapy for the rash. A punch biopsy was also performed with pathology confirming the diagnosis of acute generalized exanthematous pustulosis (Figures 3, 4, 5). 

The patient subsequently underwent surgical debridement of the left sternoclavicular joint with the placement of a wound vacuum-assisted closure device (wound vac) for the fluid collection seen on the CT scan. The head of the clavicle and left side of the manubrium were resected as both appeared necrotic with the presence of frank pus, thus confirming an abscess. 

Following surgery, the patient completed a four-week course of intravenous antibiotics with daptomycin and aztreonam. Her rash showed substantial improvement over a couple of days following cessation of cephalexin. She was discharged home in stable condition, with a follow-up plan for both dermatology and surgical assessment of the sternoclavicular joint.

## Discussion

AGEP is a rare, potentially life-threatening dermatological emergency that typically presents as an adverse drug reaction [1]. The pathophysiology of AGEP is not fully understood. Still, it is thought to involve a Type IV hypersensitivity reaction where drug antigens activate T-cells and lead to the release of pro-inflammatory cytokines along with the formation of sterile pustules [3]. AGEP most commonly manifests within 1-3 days after culprit drug exposure but can appear as late as several weeks afterward [4]. Cephalexin, a beta-lactam antibiotic frequently used to treat bacterial infections such as streptococcal pharyngitis, is a known trigger of AGEP and was identified as the likely causative agent in this case [3]. A hallmark of AGEP is the rapid resolution of the pustular rash upon withdrawal of the offending agent, typically within 15 days [5,6]. As exhibited in this case, the patient’s rash rapidly improved following discontinuation of cephalexin.

The clinical presentation of AGEP can overlap with other severe dermatologic and systemic conditions, making accurate diagnosis critical but challenging. Conditions like Stevens-Johnson syndrome, toxic epidermal necrolysis, and generalized pustular psoriasis may present with similar widespread pustular or erythematous rashes [7,8]. Distinguishing AGEP from these conditions is essential, as management approaches will differ significantly. Histologic evaluation, including the presence of subcorneal pustules and dermal neutrophilic infiltration without frank tissue necrosis, often helps confirm the diagnosis of AGEP [9,10]. Additionally, AGEP is typically associated with neutrophilia, as seen in this patient. Eosinophilia is less common but may also support the diagnosis. Early recognition of AGEP and its differentiation from other severe skin reactions allows for prompt discontinuation of the offending drug.

The sternoclavicular abscess, in this case, was a complication that added to diagnostic complexity. The patient's rash could have been initially misconstrued as being part of the presentation of a disseminated infection [11]. However, promptly considering AGEP as a differential diagnosis led to immediate cessation of the offending antibiotic. It is unlikely that the AGEP itself was the direct cause of the patient’s abscess. Instead, the abscess likely arose from the spread of infection secondary to transient bacteremia from the original streptococcal pharyngitis [12]. 

## Conclusions

The case highlights the occurrence of AGEP, triggered by a beta-lactam antibiotic, in a patient with complicated history including Crohn's disease and subsequent development of sternoclavicular joint abscess necessitating surgical intervention. AGEP, though rare, should be considered in the differential diagnosis of acute pustular eruptions, particularly in patients with recent drug exposure. Early recognition of AGEP is essential, as prompt withdrawal of the offending drug is central to achieving optimal outcomes and preventing further complications. In patients with complex medical histories, such as immunosuppressive conditions or chronic inflammatory diseases, AGEP may present alongside atypical complications, adding diagnostic and therapeutic challenges. This case underscores the value of a multidisciplinary approach, with dermatology, infectious disease, and surgical teams work together to manage both cutaneous and systemic manifestations effectively.
